# Supplementary material for: Iron and manganese co-limit the growth of two phytoplankton groups dominant at two locations of the Drake Passage
Source: Commun Biol. 2022 Mar 4;5:207. doi: 10.1038/s42003-022-03148-8 (PMC8897415; doi:10.1038/s42003-022-03148-8)
Supplement: Supplementary file 2 — Supplementary Materials [file 42003_2022_3148_MOESM2_ESM.pdf]

Supplementary Material for:

Iron and manganese co-limit the growth of two phytoplankton groups dominant at two locations of the Drake Passage.

Jenna Balaguer[1,3*], Florian Koch[2,3], Christel Hassler[4,5], Scarlett Trimborn[1,3]

[1] Marine Botany, University of Bremen, 28359 Bremen, Germany

[2]University of Applied Science Bremerhaven, 27568 Bremerhaven, Germany

[3]Ecological Chemistry, Alfred Wagener Institute Helmholtz Centre for Polar and Marine Research, 25570, Bremerhaven, Germany

[4]Swiss Polar Institute, Ecole Polytechnique Fédérale de Lausanne, Lausanne, Switzerland

[5]Department F.-A. Forel for Environmental and Aquatic Sciences, University of Geneva, Geneva, Switzerland

* jenna.balaguer@awi.de

Content

- Supplementary Methods 2
- Supplementary Tables 3
- Supplementary Figures 6
- Supplementary References 9

Supplementary Methods

Only at the end of both experiments, electron transport rates (ETR) irradiance curves were determined applying 9 different irradiances from 0 up to 1700 µmol photons m^{-2} s^{-1} with an acclimation phase of 5 min for each light level. A light sensor (ULM-500 Universal Light Meter equipped with a Spherical Micro Quantum Sensor US-SQS, Walz GmbH, Effeltrich, Germany) measured each light intensity (E, µmol photons m^{-2} s^{-1}) emitted from the FastAct Laboratory system enabling the calculation of the absolute electron transport rates (aETR, e^- $PSII^{-1}$ s^{-1}) according to the formula by Suggett et al. (2004)[1]:

$$ETR = \sigma_{PSII} \cdot \frac{\left(\frac{F'_q}{F'_m}\right)}{\left(\frac{F_v}{F_m}\right)} \cdot E$$

where F'_q/F'_m is the effective PSII quantum yield at ambient light. Maximum ETR (ETR_{max}, e^- $PSII^{-1}$ s^{-1}), light utilization efficiency (α), which is the slope of the curve at low irradiance, and minimum saturating irradiance (I_K, µmol photons m^{-2} s^{-1}) were calculated from the fitted irradiance-dependent ETR[2] (Suppl. Table 4), through a Nelder-Mead method coded on R Studio (version 1.1.463, © 2009-2016).

Supplementary Tables

Supplementary Table 1. Microscopic cell counts (cells mL^{-1}) determined of the initial community sampled at station West and East (n = 1) and at the end of experiment West (a) and East (b) after exposure to different Fe and Mn availabilities. Values represent the mean ± SD (n=3). Different letters indicate significant differences between treatments ($p < 0.05$).

	***Fragilariopsis* sp.** cells mL^{-1}	***Chaeotoceros* sp.** cells mL^{-1}	***Pseudo-nitzschia* sp.** cells mL^{-1}	***Phaeocystis antarctica*** cells mL^{-1}
**West**				
Initial	306 ~95%	6 ~2%	3 ~1%	6 ~2%
Control	589 ± 12 [c]	81 ± 16 [ab]	31 ± 2 [b]	6 ± 2 [b]
+Mn	720 ± 12 [c]	92 ± 16 [ab]	28 ± 20 [b]	2 ± 3 [b]
+Fe	1057 ± 10 [b]	112 ± 16 [a]	167 ± 15 [a]	39 ± 5 [a]
+FeMn	1429 ± 10 [a]	66 ± 16 [b]	135 ± 20 [a]	10 ± 4 [b]
**East**				
Initial	230 ~77%	43 ~14%	20 ~7%	7 ~2%
Control	442 ± 18 [b]	290 ± 17 [b]	105 ± 14 [b]	71 ± 54 [a]
+Mn	430 ± 21 [b]	190 ± 22 [c]	88 ± 35 [b]	13 ± 7 [a]
+Fe	1116 ± 217 [a]	329 ± 37 [ab]	648 ± 168 [a]	470 ± 384 [a]
+FeMn	1383 ± 178 [a]	401 ± 55 [a]	687 ± 113 [a]	205 ± 32 [a]

Supplementary Table 2. Flow cytometry cell counts (cells mL^{-1}) determined of the initial community sampled at station West and East (n = 1) and at the end of experiment West (a) and East (b) after exposure to different Fe and Mn availabilities. Values represent the mean ± SD (n=6). Different letters indicate significant differences between treatments ($p < 0.05$).

	**P1** cells mL^{-1}	**P2** cells mL^{-1}	**P3** cells mL^{-1}
**West**			
Initial	743 ~32%	1332 ~58%	222 ~10%
Control	2986 ± 633 ^{c}	918± 199 ^{b}	50 ± 16 ^{b}
+Mn	3270 ± 330 ^{bc}	828± 204 ^{b}	54 ± 26 ^{b}
+Fe	3779 ± 936 ^{ab}	1655± 429 ^{a}	342 ± 94 ^{a}
+FeMn	4092 ± 474 ^{a}	1647± 269 ^{a}	349 ± 79 ^{a}
**East**			
Initial	483 ~34%	701 ~49%	246 ~17%
Control	1928 ± 219 ^{a}	1397 ± 260 ^{c}	250 ± 40 ^{b}
+Mn	1359 ± 262 ^{b}	1415 ± 268 ^{c}	211 ± 31 ^{c}
+Fe	453 ± 70 ^{d}	3498 ± 399 ^{b}	829 ± 222 ^{a}
+FeMn	651 ± 71 ^{c}	4611 ± 747 ^{a}	882 ± 118 ^{a}

Supplementary Table 3. Concentrations of the macronutrients (NO_x=NO_3 (nitrate)+ NO_2 (nitrite), PO_4=phosphate, $SiOH_4$=silicate) were determined in each treatment at the end of the experiments West and East. Values represent the mean ± SD (n=3).

	NO_x µM	PO_4 µM	$SiOH_4$ µM
**West**			
Control	24.5 ± 0.14	1.60 ± 0.01	16.5 ± 0.18
+Mn	24.5 ± 0.17	1.55 ± 0.04	16.2 ± 0.27
+Fe	23.2 ± 0.25	1.44 ± 0.02	14.8 ± 0.31
+FeMn	23.5 ± 0.06	1.46 ± 0.06	14.7 ±0.07
**East**			
Control	24.6 ± 0.10	1.57 ± 0.02	20.6 ± 0.32
+Mn	24.8 ± 0.15	1.57 ± 0.03	21.1 ± 0.37
+Fe	22.1 ± 0.48	1.35 ± 0.02	18.2 ± 0.61
+FeMn	22.1 ± 0.51	1.34 ± 0.02	17.6 ± 0.07

Supplementary Table 4. The minimum saturating irradiance (I_k), light utilization efficiency (α) and absolute maximum electron transport rate (ETR_{max}) were determined at the end of the experiment from the communities sampled West and East after exposure to different Fe and Mn availabilities. Values represent the mean ± SD (n=3).

	I_k μmol photons m^{-2} s^{-1}	α rel.unit	ETR_{max} $e^{-}PSII^{-1}s^{-1}$
**West**			
Control	76 ± 65	2.64 ± 0.91	201 ± 69
+Mn	126 ± 111	2.43 ± 0.32	306 ± 36
+Fe	206 ± 184	2.46 ± 0.07	507 ± 13
+FeMn	107 ± 71	1.91 ± 0.13	204 ± 9
**East**			
Control	81 ± 68	2.55 ± 0.23	207 ± 16
+Mn	123 ± 113	2.97 ± 0.09	366 ± 10
+Fe	130 ± 107	1.58 ± 0.36	206 ± 38
+FeMn	113 ± 86	1.82 ± 0.13	206 ± 11

Supplementary Figures

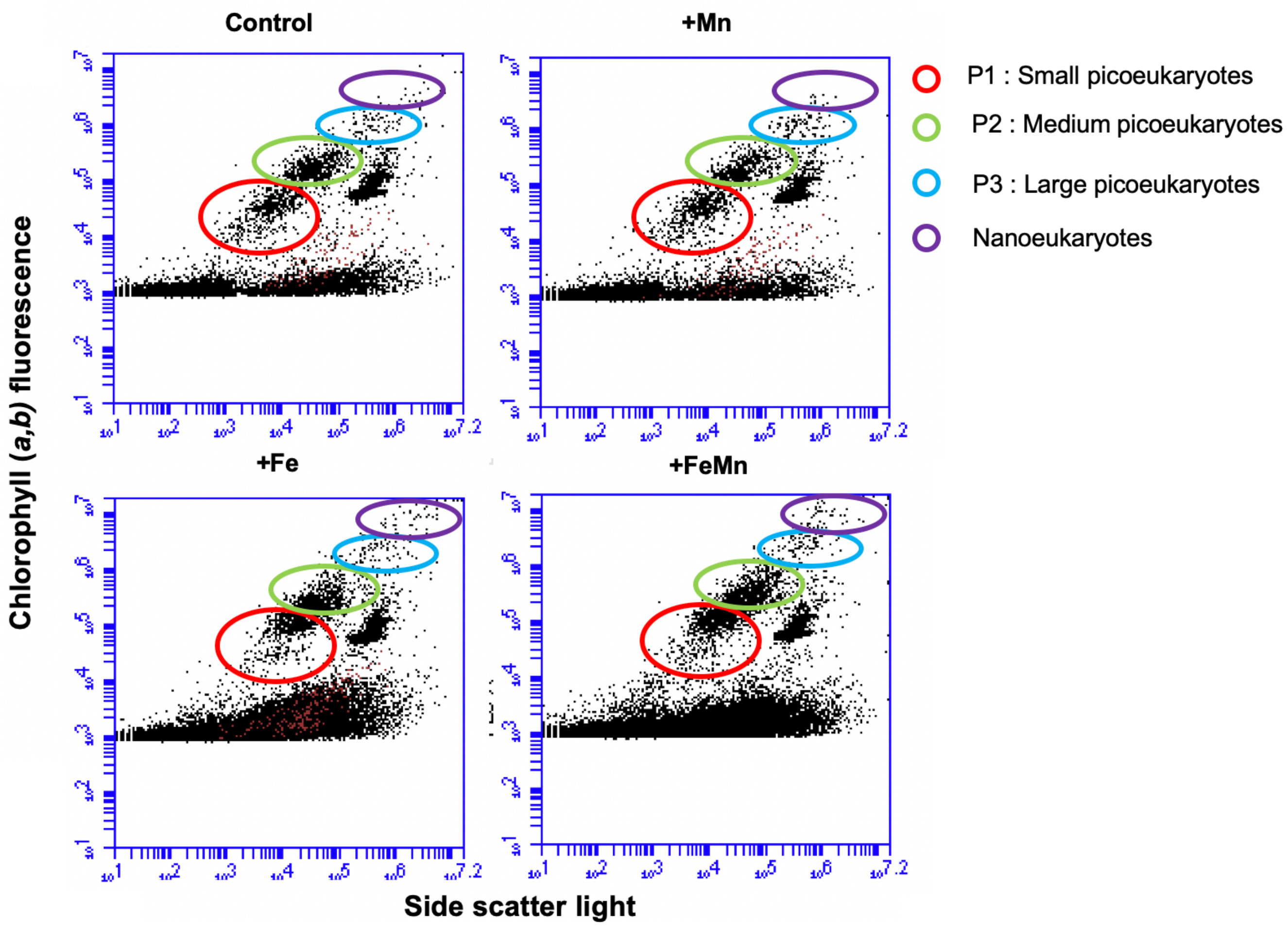

Supplementary Figure 1. Examples of flow cytometric counts and the 4 gating groups for each treatment.

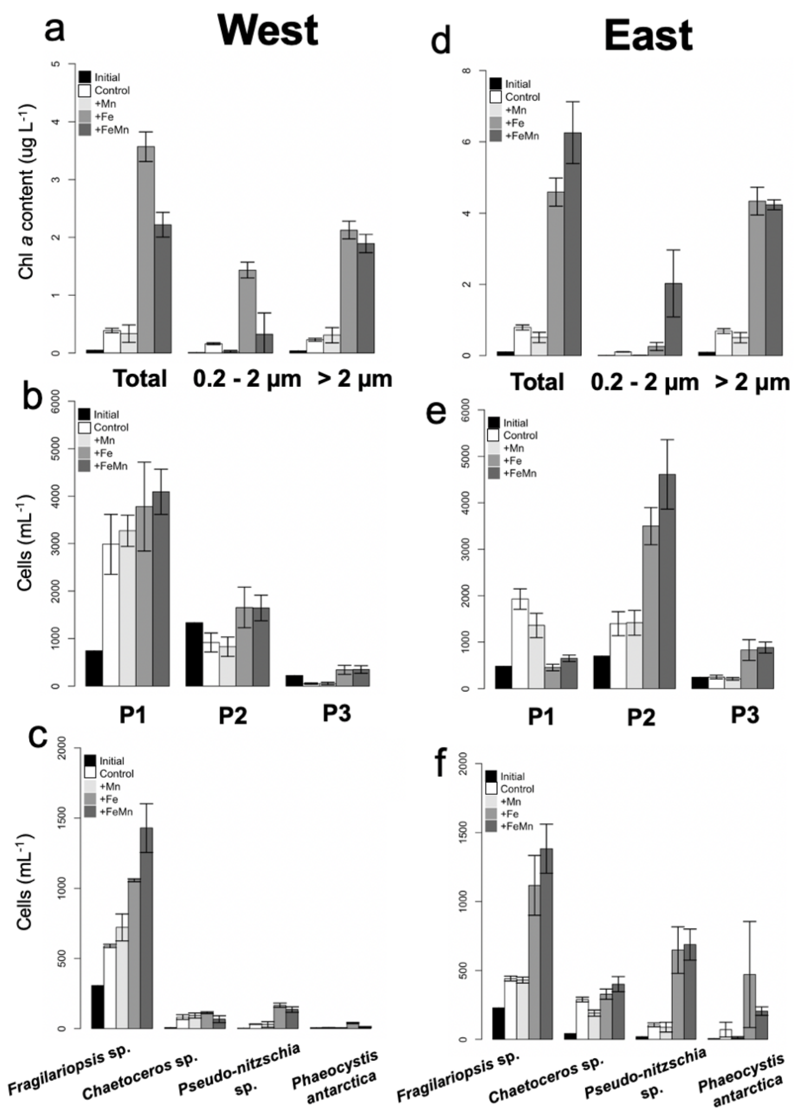

Supplementary Figure 2. Chlorophyll a content (ug L-1) and the abundances of picoeukaryotes (three subgroups P1-P3), *Chaetoceros* sp., *Fragilariopsis* sp., *Pseudo-nitzschia* sp. and *Phaeocystis antarctica* were determined at the end of the experiment for the communities sampled West (a) and East (b) after exposure to different Fe and Mn availabilities. Values represent the mean $\pm$ SD (n=3).

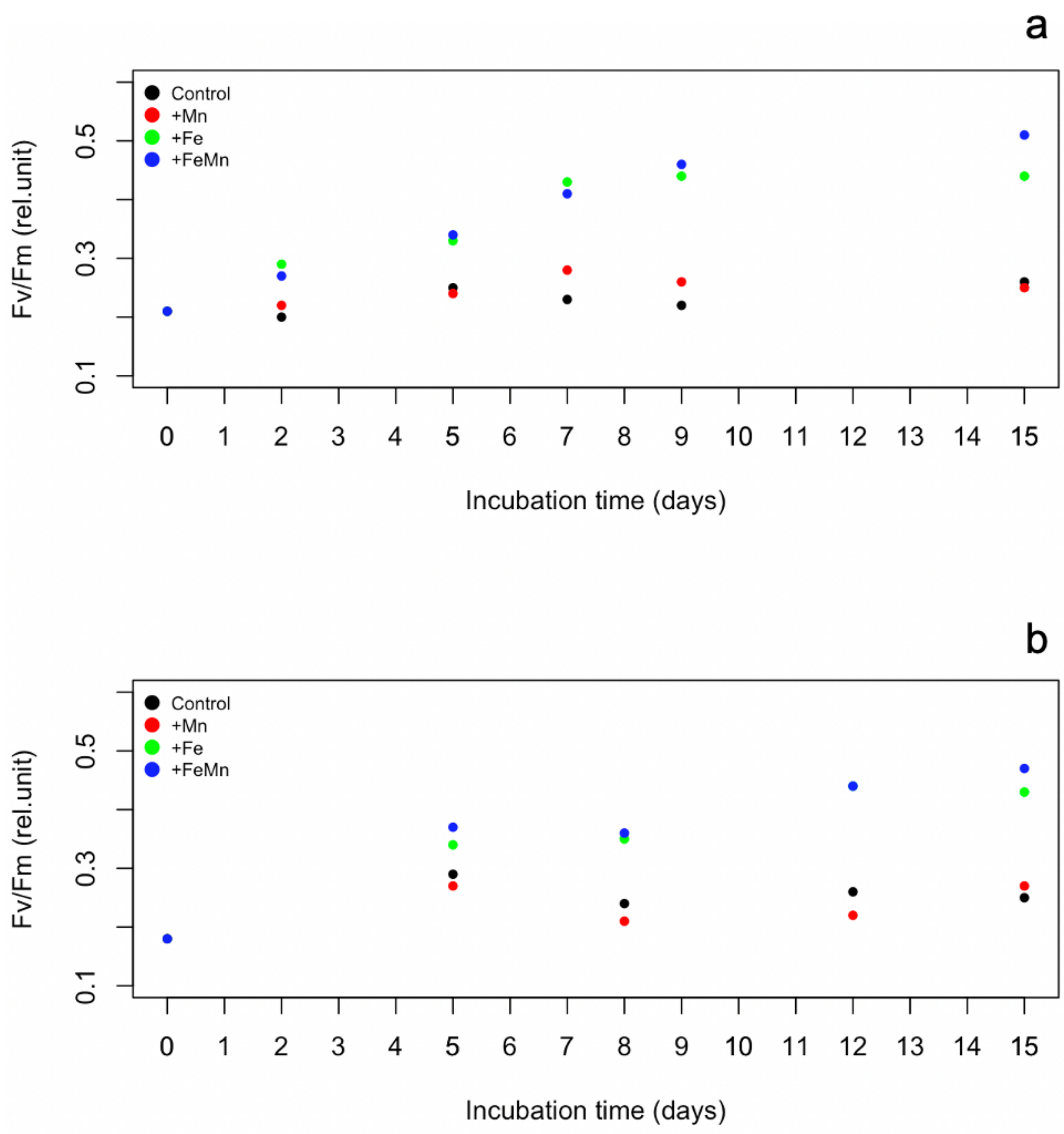

Supplementary Figure 3. The dark-adapted maximum photosystem II quantum yield (F_v/F_m) over time for experiment West (a) and East (b).

Supplementary References

1. Suggett, D. J., MacIntyre, H. L. & Geider, R. J. Evaluation of biophysical and optical determinations of light absorption by photosystem II in phytoplankton. *Limnology and Oceanography: Methods* **2**, 316–332 (2004).
2. Silsbe, G. M. & Kromkamp, J. C. Modeling the irradiance dependency of the quantum efficiency of photosynthesis. *Limnology and Oceanography: Methods* **10**, 645–652 (2012).
