# Supplementary material for: Iron and manganese co-limit the growth of two phytoplankton groups dominant at two locations of the Drake Passage
Source: Commun Biol. 2022 Mar 4;5:207. doi: 10.1038/s42003-022-03148-8 (PMC8897415; doi:10.1038/s42003-022-03148-8)
Supplement: Supplementary file 3 — Reporting Summary [file 42003_2022_3148_MOESM3_ESM.pdf]

nature research

Corresponding author(s): Jenna Balaguer

Last updated by author(s): Jan 4, 2022

Reporting Summary

Nature Research wishes to improve the reproducibility of the work that we publish. This form provides structure for consistency and transparency in reporting. For further information on Nature Research policies, see our Editorial Policies and the Editorial Policy Checklist.

Statistics

For all statistical analyses, confirm that the following items are present in the figure legend, table legend, main text, or Methods section.

n/a	Confirmed	
☐	☒	The exact sample size (*n*) for each experimental group/condition, given as a discrete number and unit of measurement
☐	☒	A statement on whether measurements were taken from distinct samples or whether the same sample was measured repeatedly
☐	☒	The statistical test(s) used AND whether they are one- or two-sided<br>*Only common tests should be described solely by name; describe more complex techniques in the Methods section.*
☒	☐	A description of all covariates tested
☒	☐	A description of any assumptions or corrections, such as tests of normality and adjustment for multiple comparisons
☐	☒	A full description of the statistical parameters including central tendency (e.g. means) or other basic estimates (e.g. regression coefficient) AND variation (e.g. standard deviation) or associated estimates of uncertainty (e.g. confidence intervals)
☒	☐	For null hypothesis testing, the test statistic (e.g. *F*, *t*, *r*) with confidence intervals, effect sizes, degrees of freedom and *P* value noted<br>*Give P values as exact values whenever suitable.*
☒	☐	For Bayesian analysis, information on the choice of priors and Markov chain Monte Carlo settings
☒	☐	For hierarchical and complex designs, identification of the appropriate level for tests and full reporting of outcomes
☐	☒	Estimates of effect sizes (e.g. Cohen's *d*, Pearson's *r*), indicating how they were calculated

Our web collection on statistics for biologists contains articles on many of the points above.

Software and code

Policy information about availability of computer code

Data collection	1. Microscopy samples were processed on a inverted light microscope (Axiovert 200; Zeiss),<br>2. Chlorophyll cell content were analyzed fluorometrically on a Trilogy Fluorometer (Turner Design, San Jose, CA, USA)<br>3. Chloropyll a fluorescence measurements were collected using a Fast Repetition Rate Fluorometer (FRRf) coupled to a FastAct Laboratory system (FastOcean PTX) and the data were derived using the FastPro8 Software (Version 1.0.55, Kevin Oxborough, CTG Ltd).<br>4. Dissolved trace metals were determined on a SeaFast system (Elemental Scientific, Omaha, NE, USA) (59,60) coupled to an inductively coupled plasma mass spectrometer (ICP-MS, Element2, Thermo Fisher Scientific, resolution of R = 2000). And the macronutrients using a QuAAtro autoanalyzer (Seal Analytical).<br>5. Flow cytometry samples were analyse with a BD Accuri™ C6 Plus flow cytometer (Becton, Dickinson and Company)
Data analysis	All analyses were performed with R Studio (version 1.1.463, © 2009-2016) and all maps with Ocean Data View (Schlitzer, 2015) .

For manuscripts utilizing custom algorithms or software that are central to the research but not yet described in published literature, software must be made available to editors and reviewers. We strongly encourage code deposition in a community repository (e.g. GitHub). See the Nature Research guidelines for submitting code & software for further information.

Data

Policy information about availability of data

All manuscripts must include a data availability statement. This statement should provide the following information, where applicable:

- Accession codes, unique identifiers, or web links for publicly available datasets
- A list of figures that have associated raw data
- A description of any restrictions on data availability

All data needed to evaluate the conclusions in the paper are present in the paper and/or Supplementary Materials and are freely available from the PANGAEA data

April 2020

repository.

Field-specific reporting

Please select the one below that is the best fit for your research. If you are not sure, read the appropriate sections before making your selection.

☐ Life sciences ☐ Behavioural & social sciences ☒ Ecological, evolutionary & environmental sciences

For a reference copy of the document with all sections, see nature.com/documents/nr-reporting-summary-flat.pdf

Ecological, evolutionary & environmental sciences study design

All studies must disclose on these points even when the disclosure is negative.

Study description

Phytoplankton were grown and incubated in 2.5 L bottles under different treatments. The 2 experiments consist of 4 treatments conducted in triplicates.

Treatment 1: Control x 3
Treatment 2: Addition of manganese x 3
Treatment 3: Addition of iron x 3
Treatment 4: Addition of iron and manganese x 3

This experiment has been performed 2 times at 2 different locations with the same abiotic parameters.

Research sample

The research samples were 2 phytoplankton communities, mainly composed by Fragilariopsis sp., Chaetoceros sp., Pseudo-nitzschia sp. and Phaeocystis antarctica. The choice of the sample research was mainly be done according to the location in the Drake Passage which reveals a high nutrient and low chlorophyll area.

Sampling strategy

The sampling strategy differed among treatments and experiments. The duration of both experiments ranged between 14-17 days according to the treatment. The physiological health of the community was assessed every 2-4 days and enable to determine the day of the harvesting (=sampling).

Data collection

The same day of the harvesting, the data collection is then performed mainly by filtration. This was done by Florian Koch and Scarlett Trimborn.

Timing and spatial scale

The duration of both experiments ranged between 14-17 days according to the treatment and the data collection was the same day as the experiment stoped. Then the samples were stored for further analysis in the laboratory at AWI Bremerhaven.

Data exclusions

No data were excluded from the analysis.

Reproducibility

The experimental setup of the experiment is very reproducible. Although, the results highly depend on the initial community of phytoplankton at a certain location. For this reason, we performed 2 times the experiment at 2 different locations, with some common results at both locations.

Randomization

Based on biogeochemical data, the experiment locations were choosen before hand and therefore not random. Although, the phytoplankton species present in the water was not known before.

Blinding

All data were acquired before any statistical analyses were run. No blinding is involved in this study.

Did the study involve field work? ☒ Yes ☐ No

Field work, collection and transport

Field conditions

Expedition aboard RV Polarstern (PS97) in 2016 in the Drake Passage (Southern Ocean).
No difficult climatic conditions during the 2 experiments performed.

Location

The expedition took place between 58° and 61° South of the Drake Passage.
Experiment 1: 60° 24.78' S 66° 21.85' W
Experiment 2: 58° 52.17' S 60° 51.92' W
The water for both experiments was taken from 25m depth.

Access & import/export

The samples were stored direclty onboard Polarstern and went back to Bremerhaven with the ship.

Disturbance

No disturbance to mentioned for this study.

Reporting for specific materials, systems and methods

We require information from authors about some types of materials, experimental systems and methods used in many studies. Here, indicate whether each material, system or method listed is relevant to your study. If you are not sure if a list item applies to your research, read the appropriate section before selecting a response.

Materials & experimental systems

n/a	Involved in the study
☒	☐ Antibodies
☒	☐ Eukaryotic cell lines
☒	☐ Palaeontology and archaeology
☒	☐ Animals and other organisms
☒	☐ Human research participants
☒	☐ Clinical data
☒	☐ Dual use research of concern

Methods

n/a	Involved in the study
☒	☐ ChIP-seq
☐	☒ Flow cytometry
☒	☐ MRI-based neuroimaging

Flow Cytometry

Plots

Confirm that:

☒ The axis labels state the marker and fluorochrome used (e.g. CD4-FITC).

☒ The axis scales are clearly visible. Include numbers along axes only for bottom left plot of group (a 'group' is an analysis of identical markers).

☒ All plots are contour plots with outliers or pseudocolor plots.

☒ A numerical value for number of cells or percentage (with statistics) is provided.

Methodology

Sample preparation

1. At the start and the end of the experiment, samples (phytoplankton in filtered sea water) were preserved with 10% buffered formalin and then Flash-frozed in liquid nitrogen.

2. Before running the samples:
For phytoplankton communities: 2 µL beads (Sperotech - Rainbow Fluorescent Particles (RFPs) - 2.11 µm) were added to each treatment as a size and fluorescence reference.
For heterotrophic bacteria: stained with 2 µL of Synergy Brands [SYBRTM] Green.

Instrument

BD Accuri™ C6 Plus flow cytometer (Becton, Dickinson and Company)

Software

Data were extracted directly from the BD Accuri™ C6 Plus software and further analysis were done with R Studio (version 1.1.463, © 2009-2016).

Cell population abundance

Abundances:
1.Heterotrophic bacteria: ~4 cells * 10-5.mL-1
2.Nanoeukaryotes: between 0-400 cells mL-1 according the the treatments
3.Picoeukaryotes: between 100-5000 cells mL-1 according the the treatments

Before starting with samples, the instrument was cleaned in order to reach 0 events per µL. In addition, two blanks with miliQ water and filtered sea water were performed, and then the blank counting could be removed from the counting of the sample.

Gating strategy

Then pico- (P), nanoeukaryotes and (N) were identified based on side scatter versus FL-3 and heterotrophic bacteria (B) on side scatter versus FL-1. Three P subgroups (0.2 – 2 µm) were differentiated according to their size: small (P1), medium (P2) and large (P3), according to sub-cluster of events, as shown in the Supplementary Fig. 1.

☒ Tick this box to confirm that a figure exemplifying the gating strategy is provided in the Supplementary Information.
